# Phytochemical Insights and Therapeutic Potential of *Chamaenerion angustifolium* and *Chamaenerion latifolium*

**DOI:** 10.3390/molecules30051186

**Published:** 2025-03-06

**Authors:** Akmaral Kozhantayeva, Zhanar Iskakova, Manshuk Ibrayeva, Ardak Sapiyeva, Moldir Arkharbekova, Yerbolat Tashenov

**Affiliations:** 1 Research Institute of New Chemical Technologies, L.N. Gumilyov Eurasian National University, Satpayev Street 2, Astana 010000, Kazakhstan; 2 Department of Chemistry, Faculty of Natural Sciences, L.N. Gumilyov Eurasian National University, Satpayev Street 2, Astana 010000, Kazakhstan; 3 Faculty of Science and Technology, Yessenov University, Aktau 130000, Kazakhstan; 4 Department of General and Biological Chemistry, NJSC “Astana Medical University”, Astana 010000, Kazakhstan

**Keywords:** *Chamaenerion angustifolium*, *Chamaenerion latifolium*, phytochemicals, antioxidant activity, antimicrobial, antiproliferative, oenothein B

## Abstract

The *Chamaenerion* genus, particularly *Chamaenerion angustifolium* and *Chamaenerion latifolium*, is recognized for its rich phytochemical composition and extensive medicinal properties. These species are abundant in polyphenols, flavonoids, and tannins, which contribute to their potent antioxidant, antimicrobial, and anticancer activities. This review provides a comprehensive analysis of their phytochemical constituents, with an emphasis on how processing methods, including fermentation, influence bioactivity. Notably, fermentation enhances the levels of key bioactive compounds, such as oenothein B, gallic acid, and ellagic acid, thereby increasing their pharmacological potential. Additionally, this review evaluates the biological activities of *Chamaenerion* species in relation to their chemical composition, while also considering the limitations of current studies, such as the lack of in vivo or clinical trials. The literature for this review was sourced from scientific databases, including PubMed, Scopus, and ScienceDirect, covering research from 2010 to 2024. Future studies should focus on optimizing extraction methods, elucidating synergistic bioactivities, and conducting in-depth clinical trials to validate their efficacy and safety.

## 1. Introduction

Therapeutic plants are of major importance in human life due to their bioactive phytochemicals, which provide potential health benefits and possess commercial value [1,2,3].

**Figure 1:**
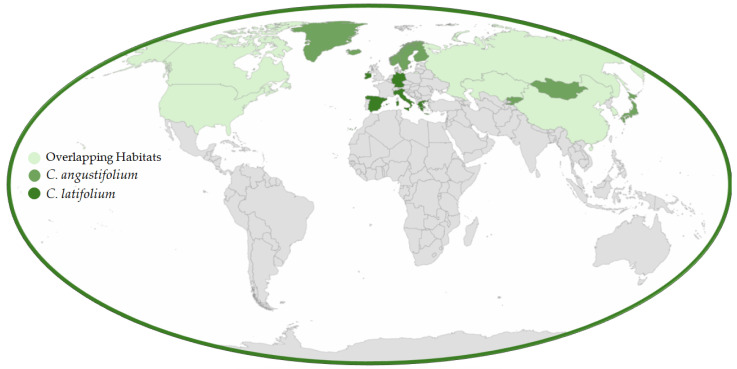
Geographical distribution of *Chamaenerion angustifolium* and *Chamaenerionlatifolium* and their overlapping habitats.

The *Onagraceae* is a large family of flowering plants with around 650 species of trees, shrubs, and herbs spread throughout approximately 17 genera [4,5]. This family is divided into two subfamilies: *Ludwigioideae*, which primarily includes the genus *Ludwigia*; and *Onagroideae*, sometimes referred to as the willowherb or evening primrose family. Many species within *Onagroideae* are known for their therapeutic and dietary benefits [6]. Characteristically, *Onagroideae* species have two to four (rarely three) deciduous sepals, while *Ludwigioideae* often have three to four persistent sepals, allowing for clear differentiation between them [4]. Several well-known garden plants belong to this family, including evening primrose (*Oenothera* L.) and fuchsia (*Fuchsia* L.). Additionally, many *Onagraceae* species are valued for their medicinal applications. Many Onagraceae species, including *Oenothera biennis*, *Epilobium angustifolium*, and *Ludwigia octovalvis*, exhibit strong antioxidant, anti-inflammatory, and antimicrobial properties due to their rich flavonoid and polyphenol content. Additionally, compounds from *Oenothera biennis* and *Oenothera paradoxa* have demonstrated cytotoxic effects against prostate and breast cancer cells [7,8,9]. Among the genera of *Onagraceae*, *Chamaenerion* (including *C. angustifolium* and *C. latifolium*) stands out for its notable medicinal properties, which are closely linked to its natural habitat and distribution. The geographical range of these species is illustrated in Figure 1.

In the last decade, the chemistry and biological activity of *Chamaenerion* species have been studied intensively, highlighting the significance of fireweed as an important medicinal plant widely utilized in the pharmaceutical, food, and cosmetic industries [10,11]. *C. angustifolium*, commonly known as willowherb or rosebay willowherb, *Chamaenerion angustifolium* (L.) *Holub*, *Chamaenerion angustifolium* (L.) *Scop.*, *Epilobium angustifolium* L., is a perennial herbaceous plant widely distributed across various habitats in the Northern Hemisphere [3,11,12]. Traditional medicine has used fireweed plants to treat a variety of ailments, such as wound healing, infections, skin diseases, colds, urinary problems like prostatitis, gastric disorders, migraine headaches, and sleep disturbances [13,14]. In northern and eastern Europe, it is utilized as a food plant, particularly in the form of tea or as a traditional herbal remedy. The widespread appeal of this plant primarily stems from its anti-inflammatory, antioxidant, antibacterial, and anticancer properties [15]. It was also reported that fireweed extract demonstrates analgesic, anticholinesterase, and skin photoprotective properties, while recent studies have emphasized its wound-healing and cosmetic benefits [16,17,18].

The therapeutic potential of *C. angustifolium* lies in its rich polyphenolic profile, particularly tannins (ellagitannins), flavonoids, and phenolic acids [19,20,21]. The principal phenolic acids include gallic acid, caffeic acid, chlorogenic acid, rosmarinic acid, ellagic acid, p-coumaric acid, and cinnamic acid, while the predominant flavonoids consist of quercetin, myricetin, kaempferol, rutin, quercetin-3-O-glucoside, and hyperoside. Oenothein B, the most abundant ellagitannin, plays a central role in the plant’s medicinal properties [22,23], exhibiting antiandrogenic, antiproliferative, anticancer, antioxidant, anti-inflammatory, and immunomodulatory activities [24,25,26]. This synergistic interaction of polyphenols and ellagitannins underscores the plant’s extensive use in traditional medicine [27,28,29]. *C. angustifolium* also contains a smallquantity of essential oil, primarily composed of terpenes, such as limonene, bisabolene, and caryophyllene, as well as eugenol, linalool, pelargol, and terpineol [30].

*C. latifolium*, commonly known as arctic fireweed, alpine fireweed, dwarf fireweed, broad-leaved fireweed, river beauty, or *Épilobe* à feuilles larges, is a long-lived perennial herb native to arctic and alpine habitats throughout the Northern Hemisphere. Its distribution includes North America, Greenland, Iceland, and northern Russia, while it is largely absent from northern Europe [31,32]. In southern Asia, it occurs in the Himalayas, ranging from Afghanistan to western China [33,34], and is also found in Central Asian regions such as Altai, Tabagatai, Dzungarian Alatau, Zailiysky Alatau, Kyrgyz Alatau, Kungei Alatau, and the Western Tien Shan (Figure 1) [20]. This species can be distinguished from its regional counterpart, *C*. *angustifolium* Holub, by its shorter, decumbent to ascending, often branched stems (up to 40 cm) and compact, few-flowered racemes [33].

*C. latifolium* is noted for its complex chemical composition, which includes bioactive compounds such as terpenes, steroids, triterpenoids, phenolic acids, and flavonoids. The key constituents are phenolic compounds, like quercetin 3-glucoside, rutin, gallic acid, caffeic acid, and chlorogenic acid, which vary based on extraction methods [35]. Ethanol extracts (ChL-EtOH) have been shown to be very rich in phenolic compounds, with strong antibacterial activity against bacterial and fungal strains, and outstanding antioxidant qualities, according to studies using HPLC-UV-ESI/MS [20]. In particular, ChL-EtOH performed best in antioxidant assessments that showed significant DPPH scavenging and FRAP capabilities. Additionally, ChL-EtOH demonstrated remarkable antibacterial activity against the fungal strain *Candida albicans* as well as Gram-positive and Gram-negative bacteria [9]. These results, together with its chemical complexity, emphasise its pharmacological potential and suggest that it shares characteristics with *C. angustifolium*, which is a rich source of bioactive compounds with interesting pharmacological significance [20].

This review aims to provide a comprehensive analysis of the botanical characteristics, chemical compositions, and biological effects of *C. angustifolium* and *C. latifolium*, exploring potential benefits for human health.

## 2. Results

### 2.1. Taxonomic Classification and Botanical Description

The taxonomic classification of *Chamaenerion* species was obtained from the World Flora Online website (https://www.worldfloraonline.org/ accessed on 24 January 2025) and is outlined in Table 1.

**Figure 2:**
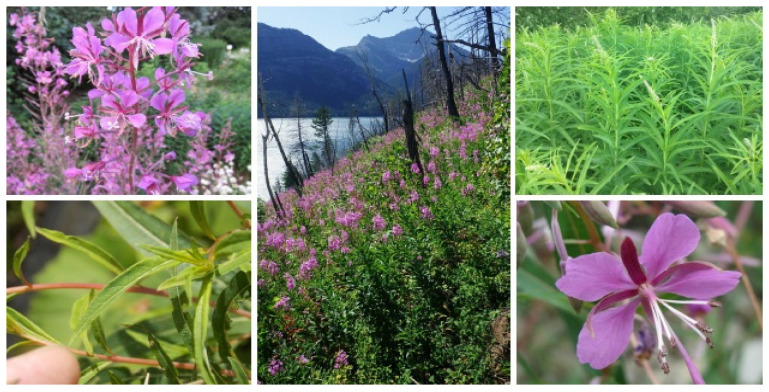
*Chamaenerion angustifolium* plant. Images retrieved with permission from https://fungi.su (accessed on 24 January 2025).

The perennial *C. angustifolium* Scop. (Figure 2) is characterized by scale-like white-pink leaves along its long rhizomes and stolons [23]. Its unbranched stems typically grow between 50 and 200 cm, either smooth or sparsely covered with short clinging hairs. The alternating leaves, densely arranged along the stem, measure 5–15 cm in length and 10–15 mm in width, featuring a linear-lanceolate shape that tapers at the base [31,32]. Dark green, glossy, and smooth, the leaves have prominent veins and margins that are either smooth or slightly serrated. As the stipules rise towards the top, they gradually become smaller, with the uppermost part often bristly [36,37,38]. The plant produces violet-pink flowers with rectangular petals, rounded at the tips, arranged in long terminal spike-like racemes. The lanceolate sepals, twice as long as the petals, are slightly hairy on the outer surface. The protandrous flowers bloom from June to September, beginning at the base of the raceme and progressing upward, with the style extending beyond the stamens [39].

**Figure 3:**
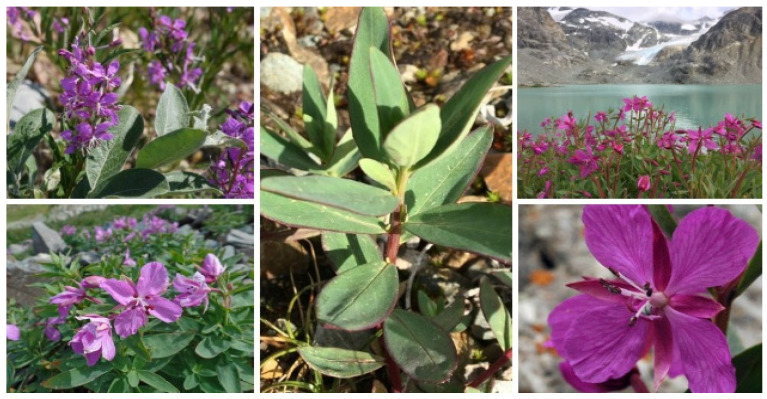
*Chamaenerion latifolium* plant. Images retrieved with permission from https://fungi.su (accessed on 24 January 2025).

*C. latifolium* (Figure 3) is a perennial plant that grows between 10 and 50 cm tall, with a thick rhizome reaching up to 1.5 cm in diameter. Its stems are branched and can be either smooth or sparsely covered with hairs, particularly near the upper part [30,31]. The leaves, which can be bare or slightly hairy, have a grayish tint. They are sessile or have very short petioles, with the lower leaves arranged oppositely and the upper leaves alternately. These leaves are thick, broadly lanceolate, and wedge-shaped at the base, blunt at the tip, and have smooth edges, measuring 2–3.5 cm in length and 1–1.5 cm in width. They are lighter on the underside and lack prominent lateral veins [40,41].

### 2.2. Phytochemicals

#### 2.2.1. Primary Metabolites

Primary metabolites (PMs) are the fundamental building blocks of life, essential for growth, development, and metabolic functions in all organisms. In plants like *C. latifolium* and *C. angustifolium*, these compounds, including carbohydrates, amino acids, fatty acids, and organic acids, are crucial for vital processes such as photosynthesis, respiration, and protein synthesis. They also serve as precursors to secondary metabolites, which contribute to the plant’s pharmacological properties [42,43]. The PMs found in *C. latifolium* and *C. angustifolium* are summarized in Table 2.

Carbohydrates are the most abundant PMs in *Chamaenerion* species, serving as the primary energy source and structural component. Glucose and galactose are particularly prominent, with glucose dominating the sugar profile. These sugars are vital for energy storage and transport, especially during flowering and seed development, reflecting the species’ adaptation to diverse environmental conditions. In *C. angustifolium*, glucose concentrations can reach up to 11.23 mg/g dry weight [44,45].

Amino acids are essential for protein biosynthesis, nitrogen transport, and stress response mechanisms. Uminska et al. analyzed the amino acid profile of *C. angustifolium* using GC-MS, identifying L-alanine (2.350–6.090 mg/g) and L-phenylalanine as the dominant amino acids, with moderate amounts of L-leucine, L-isoleucine, and L-valine. Interestingly, sulfur-containing amino acids are absent, potentially impacting the synthesis of specific sulfur-rich secondary metabolites. This strategic allocation of amino acids highlights their diverse structural and functional roles [46,47].

Fatty acids contribute to membrane integrity and bioactive lipid functions. Key compounds in *Chamaenerion* include linoleic acid, palmitic acid [48], and n-hexadecenoic acid [35]. The balanced fatty acid composition observed in both species underscores their adaptability to various ecological niches and provides precursors for secondary metabolites like lipophilic antioxidants.

**Table 2:** Primary metabolites identified in the aerial parts of *C. angustifolium* and *C. latifolium*.

Compounds	Molecular Weight, g/mol	Plant	Identification Method	Extraction Method	Extract Type	Ref.
Fatty acids						
n-Hexadecanoic	256.43	*CL*	GC-MS	Maceration	Hexane	[35]
Tetradecanoic	228.37	*CL*	GC-MS	Maceration	Hexane	[35]
Linoleic	280.45	*CA*	GC-MS	Maceration	Methanol	[35]
Palmitic	256.43	*CA*	C-MS	Reflux	Methanol	[44]
Capric	172.26	*CA*	C-MS	Reflux	MTBE	[44]
Myristic	228.37	*CA*	C-MS	Reflux	MTBE	[44]
Lauric	200.32	*CA*	C-MS	Reflux	MTBE	[44]
Pentadecanoic	242.41	*CA*	C-MS	Reflux	MTBE	[44]
Pentadecenic	240.39	*CA*	C-MS	Reflux	MTBE	[44]
Palmitoleic	254.41	*CA*	C-MS	Reflux	MTBE	[44]
Margaric	270.46	*CA*	C-MS	Reflux	MTBE	[44]
γ-Linolenic	278.43	*CA*	C-MS	Reflux	MTBE	[44]
Nonadecanoic	298.5	*CA*	C-MS	Reflux	MTBE	[44]
Tetracosanic	368.63	*CA*	C-MS	Reflux	MTBE	[44]
Heneicosanic	326.57	*CA*	C-MS	Reflux	MTBE	[44]
2-Hydroxyoctacosanic	440.74	*CA*	C-MS	Reflux	MTBE	[44]
2-Hydroxytriacontanic	468.78	*CA*	C-MS	Reflux	MTBE	[44]
Hexadecandioic	286.35	*CA*	C-MS	Reflux	MTBE	[44]
Octadecanedioic	314.47	*CA*	C-MS	Reflux	MTBE	[44]
Eicosandioic	342.52	*CA*	C-MS	Reflux	MTBE	[44]
Hexacosanic	394.66	*CA*	C-MS	Reflux	MTBE	[44]
2-Hydroxyhexacosanic	410.68	*CA*	C-MS	Reflux	MTBE	[48]
2-Hydroxytetracosanic	382.63	*CA*	C-MS	Reflux	MTBE	[44]
2-Hydroxytricosanic	368.61	*CA*	C-MS	Reflux	MTBE	[44]
Pentacosanic	396.66	*CA*	C-MS	Reflux	MTBE	[44]
Triacontanic	452.79	*CA*	C-MS	Reflux	MTBE	[44]
Octacosanic	424.73	*CA*	C-MS	Reflux	MTBE	[44]
Nonacosanic	438.76	*CA*	C-MS	Reflux	MTBE	[44]
Heptacosanic	410.71	*CA*	C-MS	Reflux	MTBE	[44]
Behenic	340.57	*CA*	C-MS	Reflux	MTBE	[44]
Arachic	312.52	*CA*	C-MS	Reflux	MTBE	[44]
Tricosanic	366.64	*CA*	C-MS	Reflux	MTBE	[44]
Amino acids						
L-Alanine	89.09	*CA*	GC-MS,	SPE	Methanol	[47,48]
L-Phenylalanine	165.19	*CA*	GC-MS	UAE	Methanol	[47,48]
L-Leucine	131.18	*CA*	GC-MS	UAE	Methanol	[47,48]
L-Isoleucine	131.18	*CA*	GC-MS	UAE	Methanol	[47,48]
L-Proline	115.13	*CA*	GC-MS	SPE	Methanol	[48]
L-Serine	105.09	*CA*	GC-MS	SPE	Methanol	[48]
L-Threonine	119.12	*CA*	GC-MS	SPE	Methanol	[48]
L-Phenylalanine	165.19	*CA*	GC-MS	SPE	Methanol	[48]
L-Aspartic acid	133.1	*CA*	GC-MS	SPE	Methanol	[48]
L-Glutamic acid	147.13	*CA*	GC-MS	SPE	Methanol	[48]
Carbohydrates						
D-Glucose	180.16	*CA*	GC-MS	SPE	Methanol	[48]
D-Galactose	180.16	*CA*	GC-MS	SPE	Methanol	[48]
Myo-Inositol	180.16	*CA*	GC-MS	Reflux	Methanol	[44]
D-Mannose	180.16	*CA*	GC-MS	Reflux	Methanol	[44]
D-Arabinose	150.13	*CA*	GC-MS	Reflux	Methanol	[44]
D-Ribose	150.13	*CA*	GC-MS	Reflux	Methanol	[44]
Glucose	180.16	*CL*	PC	Maceration	Aqueous	[45]
Galactose	180.16	*CL*	PC	Maceration	Aqueous	[45]
Xylose	150.13	*CL*	PC	Maceration	Aqueous	[45]

MTBE—Methyl tert-Butyl Ether; *CL*—*C. latifolium*; *CA*—*C. angustifolium*; PC—paper chromatography; SPE—stepwise percolation extraction; UAE—ultrasonic-assisted extraction.

#### 2.2.2. Volatile and Lipophilic Constituents

Volatile and lipophilic compounds are integral to plants, contributing to their unique scents, facilitating ecological interactions, and enhancing their medicinal properties [49,50]. In *C. angustifolium* and *C. latifolium*, these compounds play a vital role in antioxidant, antimicrobial, and anti-inflammatory activities, making them a central focus in pharmacological research [9]. Advanced techniques, like gas chromatography–mass spectrometry (GC-MS), have allowed for the detailed exploration of these bioactive compounds, revealing their diversity and functional significance [51]. A detailed volatile and lipophilic contents of both plants islisted in Table 3.

The lipophilic fraction of *Chamaenerion* species is notably rich in long-chain hydrocarbons, esters, and triterpenoids. GC-MS analysis of hexane extracts from *C. latifolium* highlights a substantial presence of these classes of compounds. Among them, nonacosane and tetracosanol dominate as the primary alkanes and alcohols, accounting for 31.339% of the leaves and 48.158% of the stems. These long-chain hydrocarbons possess hydrophobic properties, making them valuable in promoting a skin barrier function in medicinal applications [35]. In *C. angustifolium*, pentacosanal stands out as a significant aldehyde, contributing 31.1% of the aldehyde fraction. This long-chain aldehyde is linked to anti-inflammatory properties [52].

Volatile compounds impart *Chamaenerion* species with their distinctive aromas, which play a crucial ecological role in attracting pollinators and deterring pests. Key volatiles identified in *C. angustifolium* include trans-2-hexenal, α-pinene, and linalool. Beyond their aromatic properties, these compounds exhibit noteworthy biological activities. Trans-2-hexenal, known for its fresh, green scent, enhances the plant’s defense mechanisms and demonstrates antimicrobial effects. Similarly, α-pinene and linalool are recognized for their anti-inflammatory and antioxidant properties, underscoring their therapeutic potential [23,30]. Cis-3-hexenol, often referred to as “leaf alcohol”, is particularly abundant in fresh samples, comprising 17.5% to 68.6% of the total volatiles [23]. Additionally, sesquiterpenes, such as α- and β-caryophyllenes, are prominent for their anti-inflammatory and anticancer activities, with α-caryophyllene contributing up to 52.3% of the total volatiles in *C. angustifolium* [53].

GC-MS has revolutionized the study of volatile and lipophilic compounds, enabling their precise identification and quantification [54]. Researchers employ various extraction methods, including maceration, percolation, and hydrodistillation, to isolate these compounds from *Chamaenerion* species. Non-polar solvents like hexane and methyl tert-butyl ether are highly effective for extracting lipophilic compounds, whereas hydrodistillation is ideal for volatile aromatics [55,56]. These methodological advancements have ensured the accurate characterization of the complex chemical profiles of *Chamaenerion* species.

The therapeutic potential of volatile and lipophilic compounds in *Chamaenerion* species is substantial. Alkanes like nonacosane and tetracosane enhance the skin barrier function, making them valuable in dermatological applications [57]. Meanwhile, sesquiterpenes, including α- and β-caryophyllenes, exhibit potent anti-inflammatory and anticancer activities, with α-caryophyllene constituting a significant portion of the total volatiles [58]. These findings highlight the importance of *Chamaenerion* species as a natural source of bioactive compounds with diverse pharmacological applications.

**Table 3:** Volatile and lipophilic components of *C. angustifolium* and *C. latifolium* identified using GC-MS.

Compounds	Molecular Weight, g/mol	Plant	Plant Part	Extraction Method	Extract Type	Ref.
Sesquiterpene						
Caryophyllenes (α)	893.51	*CA*	Leaves	SPME	Methanol (aq.)	[23,53]
Caryophyllenes (β)	907.49	*CA*	Leaves	SPME,	Methanol (aq.)	[23,53]
Phenylpropanoids						
Anethole	148.20	*CA*	Leaves	SPME	Methanol (aq.)	[23,53]
Monoterpene Hydrocarbon						
α-Pinene	136.23	*CA*	Flowers	Hydrodistillation	EO	[30]
Camphene	136.23	*CA*	Flowers	Hydrodistillation	EO	[30]
Linalyl propionate	210.31	*CA*	Flowers	Hydrodistillation	EO	[30]
Terpineol	154.25	*CA*	Flowers	Hydrodistillation	EO	[30]
Oxygenated Monoterpene						
Linalool	154.25	*CA*	Flowers	Hydrodistillation	EO	[30]
Eugenol	164.20	*CA*	Flowers	Hydrodistillation	EO	[30]
Alkanes						
Tricosane	324.63	*CA*	Aerial parts	Percolation	Lipophilic	[52]
Tetradecane	198.39	*CA*	Aerial parts	Percolation	Lipophilic	[52]
Hexadecane	226.44	*CA*	Aerial parts	Percolation	Lipophilic	[52]
Heptadecane	240.47	*CA*	Aerial parts	Percolation	Lipophilic	[52]
Pentadecane	212.42	*CA*	Aerial parts	Percolation	Lipophilic	[52]
Tetracosane	338.65	*CL*	Leaves and Stems	Maceration	Hexane	[35]
Pentacosane	352.69	*CL*	Leaves and Stems	Maceration	Hexane	[35]
Hexacosane	366.70	*CL*	Leaves and Stems	Maceration	Hexane	[35]
n-Octacosane	394.77	*CL*	Leaves and Stems	Maceration	Hexane	[35]
Nonacosane	408.60	*CL*	Leaves and Stems	Maceration	Hexane	[35]
Hentriacontane	436.85	*CL*	Leaves and Stems	Maceration	Hexane	[35]
Ester						
β-Amyrenyl acetate	468.80	*CL*	Leaves and Stems	Maceration	Hexane	[35]
Icosylhexadecanoate	536.96	*CL*	Leaves and Stems	Maceration	Hexane	[35]
Bis(2-ethylhexyl) phthalate	390.55	*CL*	Leaves and Stems	Maceration	Hexane	[35]
Alcohols						
n-Tetracosanol-1	354.65	*CL*	Leaves and Stems	Maceration	Hexane	[35]
Cis-3-Hexenol	100.16	*CA*	Aerial parts	SPME	Methanol (aq.)	[23]
Aldehydes						
Nonacosanal	422.77	*CL*	Leaves and Stems	Maceration	Hexane	[35]
Pentacosanal	366.66	*CA*	Aerial parts	Percolation	Lipophilic	[52]
Tricosanal	338.60	*CA*	Aerial parts	Percolation	Lipophilic	[52]
Trans-2-Hexenal	98.14	*CA*	Leaves	SPME	Methanol (aq.)	[23]
Benzacetaldehyde	120.15	*CA*	Flowers	Hydrodistillation	EO	[30]
Triterpenoids						
α-Amyrin	426.72	*CL*, *CA*	Leaves	Maceration	Hexane	[35,52]
β-Amyrenol	426.72	*CL*, *CA*	Steams	Maceration	Hexane	[35,52]

SPME—solid-phase microextraction; *CL*—*C. latifolium*; *CA*—*C. angustifolium*; EO—essential oil.

#### 2.2.3. Polyphenolic Compounds

Polyphenolic compounds are a diverse group of secondary metabolites known for their potent antioxidant, anti-inflammatory, and anticancer properties [59,60]. These compounds, including phenolic acids, flavonoids, and tannins, play a critical role in the therapeutic potential of plants. In *C. angustifolium* and *C. latifolium*, polyphenols are abundant and diverse, contributing significantly to the pharmacological activities of these species [61]. Advanced analytical techniques, such as high-performance liquid chromatography (HPLC) coupled with UV detection, diode array detection, and multi-stage mass spectrometry, have enabled precise profiling of these bioactive compounds, providing valuable insights into their biological roles and variations under different conditions [62].

**Figure 4:**
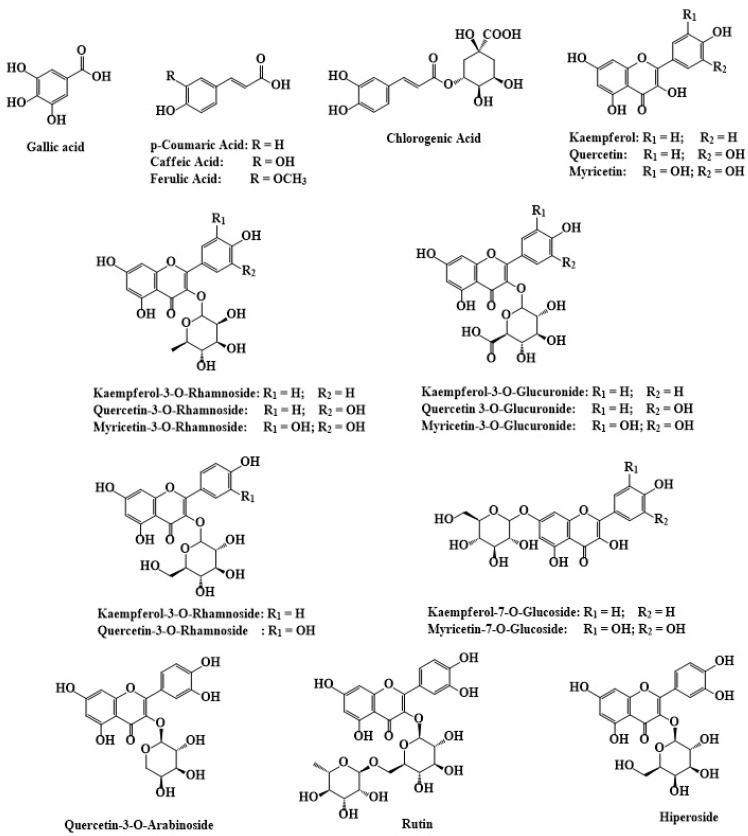
The chemical structures of polyphenolic compounds identified in *C. angustifolium* and *C. latifolium*. The structures were drawn using ChemDraw Ultra 12.0 software.

As summarized in Table 4 and Figure 4, the polyphenolic composition of *C. angustifolium* and *C. latifolium* includes key compounds, such as oenothein B, quercetin, chlorogenic acid, caffeic acid, ellagic acid, and gallic acid, which contribute significantly to their antioxidant and anti-inflammatory activities [30].

The composition and concentration of polyphenols are influenced by various factors including the growth stage, environmental conditions, and extraction methods [63,64]. Research by Gryszczyńska et al. on *C. angustifolium* demonstrated that bioactive compound concentrations vary depending on harvest time, with oenothein B, sterols, flavonoids, and polyphenolic acids reaching higher levels during the flowering period. Similarly, their study identified phenolic metabolites, with gallic acid, oenothein B, and quercetin 3-O-arabinoside as the dominant compounds. These results highlight the crucial role of harvest timing in maximizing the medicinal value of *C. angustifolium* cultivated ex vitro [65]. Further elucidating the polyphenolic profile, Maruška et al. (2014) investigated the flavonoid composition and antioxidant activity of *C. angustifolium* across different vegetation stages, applying HPLC with UV detection and DPPH radical scavenging analysis, identifying key flavonoids such as hyperoside, myricetin, quercetin, quercetin-3-O-arabinoside, myricetin-7-O-glucoside, kaempferol, kaempferol-7-O-glycosides, and kaempferol-3-O-glucoside [66]. The flavonoid concentration and antioxidant activity peaked during massive blooming, correlating with high levels of myricetin, its glycosides, and hyperoside, highlighting blooms as primary flavonoid storage sites. This phase also enhances the medicinal potential of key flavonoids, such as quercetin (antioxidant, anti-inflammatory, cardioprotective, and anticancer), kaempferol (antiproliferative and pro-apoptotic), myricetin (antibacterial), and kaempferol-7-O-glucoside (antiviral against HIV-1), emphasizing the importance of selective harvesting at full bloom for the optimal yield [65,66,67]. Recent studies highlight the biological significance of isocoumarins, structural isomers of key flavonoids like quercetin, kaempferol, and myricetin. Ramanan et al. found that 3-aryl isocoumarins inhibit 5-LOX and mPGES1, demonstrating strong anti-inflammatory activity [68]. Their structural similarity to flavonoids suggests potential synergies, warranting further research.

**Table 4:** HPLC profile of polyphenolic compounds in the aerial parts of *C. angustifolium* and *C. latifolium*.

Compounds	Molecular Weight, g/mol	Plant	Identification Method	Extraction Method	Extract Type	Ref.
Phenolic acids						
Gallic acid	170.12	*CA* *CL*	HPLC-DAD-MSn, HPLC-UV, HPLC-UV-ESI/MS	Hydrodistillation, Reflux	Methanol (aq.), Methanol, Ethanol	[9,30,53]
Chlorogenic acid	354.31	*CL*	HPLC-UV, HPLC-DAD, HPLC-UV-ESI/MS	Hydrodistillation, Reflux	Methanol (aq.), Methanol, Ethanol	[9,30,53]
Caffeic Acid	180.16	*CL*	HPLC-UV-ESI/MS	Reflux	Ethanol	[9]
Ellagic Acid	302.20	*CA*	HPLC-UV-ESI/MS	UAE	Methanol	[67]
p-Coumaric Acid	164.04	*CL*	HPLC-UV-ESI/MS	Reflux	Ethanol	[9]
Ferulic Acid	194.18	*CA*	HPLC-UV-ESI/MS	UAE	Methanol	[67]
Flavonoids						
Rutin	610.52	*CA*, *CL*	HPLC-UV, HPLC-UV-ESI/MS	UAE, Reflux	Methanol (aq.), Ethanol	[9,67]
Quercitin	302.24	*CL*, *CA*	HPLC-UV	Reflux, SPME	Methanol (aq.)	[23,66]
Quercetin-3-O-Glucoside	464.38	*CL*	HPLC-UV-ESI/MS	Reflux	Ethanol	[9]
Quercetin-3-O-Arabinoside	434.35	*CA*	HPLC-DAD-MSn	Hydrodistillation	Methanol	[66]
Quercetin 3-O-Glucuronide	478.36	*CL*, *CA*	HPLC-DAD, UPLC-MS/MS	SPE, Reflux	Methanol	[30,53,65]
Quercetin-3-O-Rhamnoside	448.38	*CA*	HPLC-DAD, HPLC-MS/MS	UAE,	Methanol	[67]
Myricetin	318.24	*CL*, *CA*	HPLC-UV-ESI/MS, HPLC-UV	Reflux, SPME	Ethanol, Methanol (aq.)	[9,23]
Myricetin-3-O-Rhamnoside	464.38	*CA*	HPLC-UV-ESI/MS	UAE	Methanol	[67]
Myricetin-3-O-Glucuronide	494.36	*CA*	HPLC-UV-ESI/MS	UAE	Methanol	[67]
Myricetin-7-O-Glucoside	480.38	*CA*	HPLC-DAD-MSn	UAE	Methanol	[67]
Kaempferol	286.24	*CL*	HPLC-UV	SPME	Methanol (aq.)	[23]
Kaempferol-3-O-Glucuronide	462.36	*CA*	HPLC-UV-ESI/MS	UAE	Methanol	[67]
Kaempferol-3-O-Rhamnoside	432.38	*CA*	HPLC-UV-ESI/MS	UAE	Methanol	[65]
Kaempferol-7-O-Glucoside	448.38	*CA*	HPLC-DAD-MSn	SPE	Methanol	[67]
Kaempferol-3-O-Glucoside	448.48	*CA*	HPLC-DAD-MSn	SPE	Methanol	[66]
Hyperoside	464.38	*CL*	HPLC-UV	SPME	Methanol (aq.),	[23]
Tannins						
Oenothein B	1569.10	*CA*	HPLC-DAD-MSn, HPLC-DAD, HPLC-UV	SPE, UAE, SPME,	Methanol, Methanol (aq.)	[23,65,67]

*CL*—*C. latifolium*; *CA*—*C. angustifolium*; SPME—solid-phase microextraction; UAE—ultrasonic-assisted extraction; SPE—solid-phase extraction.

In alignment with these findings, Kaškonienė et al. conducted a detailed HPLC analysis of polyphenolic compounds in *C. angustifolium*, focusing on the effects of drying [23]. Oenothein B was identified as the primary polyphenolic compound, abundant in both fresh and dried samples, but drying reduced its concentration approximately five-fold. Notably, Oenothein B exhibits a range of biological activities, including anti-tumor potential, the ability to decrease tumor growth in vivo, and macrophage activation. Additionally, it demonstrates anti-HIV, anti-inflammatory, antiprostate hyperplasia, and immunomodulatory properties [65]. Clinical studies have confirmed its therapeutic potential. A randomized, double-blind, placebo-controlled trial on *Epilobium angustifolium* extract (500 mg daily for six months) demonstrated significant improvements in symptoms of benign prostatic hyperplasia, including reduced post-void residual urine volume and nocturia, with good tolerability [69]. Another 12-week clinical trial in Japan showed that eucalyptus extract containing oenothein B significantly reduced the visceral fat area, waist circumference, body weight, and BMI in overweight individuals compared to a placebo [70]. Rutin levels similarly decreased by 2.2 times with drying, while quercetin and gallic acid showed stability [67]. Rutin is highly significant in scientific research due to its extensive pharmacological potential. Numerous reviews have highlighted its diverse bioactivities, including anti-inflammatory, antidiabetic, cardiovascular, hepatoprotective, anticancer, and neuroprotective effects. Additionally, glycosylated isocoumarins, which are structural isomers of rutin, have been synthesized for similar bioactive properties. Aidhen and Kasireddy synthesized 3-glycosylated isocoumarins using Julia olefination and Meinwald rearrangement, demonstrating their relevance as bioactive flavonoid analogs [12,20,70,71]. Chlorogenic acid, however, demonstrated sensitivity to preparation, as it was absent in some dried samples, indicating drying’s impact on specific polyphenols and suggesting fresh samples retain a more robust polyphenolic profile [66,67]. Chlorogenic acid possesses strong antioxidant properties and has been studied for its protective effects against UV-induced skin damage [17].

The efficient extraction of polyphenols depends on the choice of solvent and method. Ethanol has been identified as the most effective solvent for isolating polyphenols from *C. latifolium* species, yielding high concentrations of gallic acid, quercetin 3-glucoside, and rutin. By contrast, ethyl acetate extracts tend to favor selective isolation of specific compounds like myricetin, albeit at lower overall yields [9]. Advances in extraction technology, such as ultrasound-assisted extraction and solid-phase microextraction, have further enhanced the recovery and analysis of these bioactive compounds, paving the way for their utilization in pharmacological applications [72].

The polyphenolic composition of *C. angustifolium* and *C. latifolium* underscores their significance as a natural source of bioactive compounds. Seasonal and environmental factors, along with extraction methods, profoundly influence the yield and efficacy of these compounds [73].

#### 2.2.4. Impact of Fermentation on the Chemical Composition of *C. angustifolium*

Fermentation is a widely recognized process in food and pharmaceutical industries, known for enhancing the bioavailability and functionality of bioactive compounds in plant materials [74]. In the case of *C. angustifolium*, fermentation significantly alters its chemical profile, particularly increasing the concentration of polyphenols, flavonoids, and specific antioxidants [21].

The chemical composition of non-fermented and fermented *C. angustifolium* leaves highlights the significant impact of fermentation on bioactive compound concentrations (Table 5). Jarine et al. observed a significant increase in total polyphenolic content throughout both aerobic and anaerobic fermentation, peaking after 48 h of aerobic fermentation. Ellagic acid is the primary phenolic acid in rosebay willowherb leaves, with its content significantly increasing after 48 h of aerobic solid-state fermentation, escalating from 1246.56 mg to 2588.25 mg per 100 g dry weight in the fermented samples [75]. During fermentation, p-coumaric acid exhibited a decrease after 24 and 48 h of aerobic fermentation but showed a significant increase after 72 h compared to unfermented leaves. Gallic acid concentrations in non-fermented leaves started at 29.14 mg/100 g DW and rose sharply during fermentation, especially in aerobic conditions, to reach 135.20 mg/100 g DW after 24 h [76]. Lasinkas and his team reported that solid-state fermented leaves had elevated levels of benzoic acid, quercetin, and oenothein B, with oenothein B steadily increasing over two years of fermentation. A particularly noticeable rise in oenothein B was recorded after 24 h of fermentation [77].

The physiological activity of fireweed leaves is shaped by various factors, including solid-state fermentation and agricultural practices such as natural, organic, and biodynamic methods [78]. Biodynamic farming enhances soil and plant health through fermented preparations. Organic farming relies on natural compost and excludes synthetic inputs, while natural farming minimizes interventions like compost or preparations [79]. Among these practices, non-fermented samples showed the highest chlorogenic acid content (47.37 mg/100 g DW), while organic leaves achieved the peak concentration of quercetin-3-O-rutinoside (79.19 mg/100 g DW). Biodynamic farming excelled in producing the highest levels of lutein and beta-carotene, at 35.59 and 15.90 mg/100 g DW, respectively [80].

Carotenoids, vital for human health and abundant in plant-based foods, are highly recommended for inclusion in the daily diet. However, chlorophyll A and B degrade during fermentation due to oxidative or enzymatic processes, with chlorophyll B declining from 172.43 to 156.98 mg/100 g DW after 24 h, highlighting the impact of fermentation on pigment stability [80].

Moreover, proteins and fibers exhibit a modest increase with fermentation, especially after 48 h. This may result from structural changes in plant cells, enhancing digestibility. Non-fermented leaves contain higher sugar levels (7.08 mg/100 g DW). Fermentation significantly reduces the sugar content (4.23 mg/100 g DW after 48 h), as sugars are metabolized by microbes, indicating fermentation’s potential for reducing caloric content. Non-fermented leaves contain moderate levels of vitamin C (247.19 mg/100 g DW). Fermented samples showed a sharp increase in these levels (534.70 mg/100 g DW after 48 h), likely due to microbial synthesis or better preservation under acidic conditions [81].

Fermentation profoundly impacts the chemical composition of *C. angustifolium* leaves, enhancing the concentration of polyphenols, flavonoids, and specific antioxidants while reducing the sugar levels. These transformations not only improve the nutritional value of the leaves but also amplify their medicinal potential [81,82].

**Table 5:** Changes in the chemical composition of *C. angustifolium* during fermentation.

Classes	Components	Fermentation Status	Time (h)	Concentration (mg/100 g DW)	Ref.
Phenolic Acids	Gallic Acid	Non-Fermented	0	29.14	[76]
		Fermented(Aerobic)	24	135.20	[76]
	Chlorogenic Acid	Non-Fermented	0	56.79	[80]
		Fermented (natural)	24	47.37	[80]
	p-Coumaric Acid	Non-Fermented	0	213.81	[76]
		Fermented (Anaerobic)	72	255.73	[76]
	Ellagic Acid	Non-Fermented)	0	1246.56	[75]
		Fermented (Aerobic	48	2588.25	[75]
	Benzoic Acid	Non-Fermented	0	3.00	[77]
		Fermented	48	29.81	[77]
Tannins	Oenothein B	Non-Fermented	0	1442.22	[77]
		Fermented	24	1753.65	[77]
Flavonoids	Myricetin	Non-Fermented	0	11.31	[75]
		Fermented (Aerobic)	48	25.83	[75]
	Quercetin-3-O-Rutinoside	Non-Fermented	0	20.85	[80]
		Fermented (organic)	24	79.19	[80]
	Quercetin-3-O-Glucoside	Non-Fermented	0	55.61	[80]
		Fermented	24	66.20	[80]
	Quercetin	Non-Fermented	0	2.45	[21]
		Fermented	24	10.65	[21]
	Luteolin	Non-Fermented	0	6.33	[77]
		Fermented	24	2.40	[77]
	Kaempferol	Non-Fermented	0	3.87	[77]
		Fermented	24	2.70	[77]
Carotenoids	Lutein	Non-Fermented	0	33.16	[80]
		Fermented (Biodynamic)	24	35.59	[80]
	Zeaxanthin	Non-Fermented	0	14.89	[80]
		Fermented	48	17.66	[80]
	Beta-Carotene	Non-Fermented)	0	15.28	[80]
		Fermented (Biodynamic	24	15.90	[80]
Chlorophylls	Chlorophyll B	Non-Fermented	0	172.43	[80]
		Fermented	24	156.98	[80]
	Chlorophyll A	Non-Fermented	0	153.97	[80]
		Fermented (Biodynamic)	24	127.33	[80]
Carbohydrates	Total Sugars	Non-Fermented	0	7.08	[81]
		Fermented	48	4.23	[81]
Organic acids	Vitamin C	Non-Fermented	0	247.19	[81]
		Fermented	48	534.70	[81]

#### 2.2.5. Sterols

**Figure 5:**
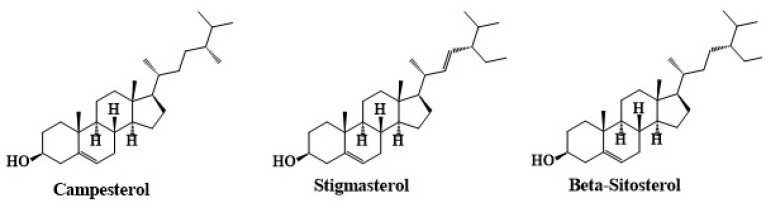
The chemical structures of sterols identified in *C. angustifolium*. The structures were drawn using ChemDraw Ultra 12.0 software.

Sterols are a vital component of the lipophilic extracts in *C. angustifolium*, showcasing significant bioactive properties and medicinal potential [83]. Analytical techniques, such as GC-MS and HPLC-DAD-MS/MS, have enabled the comprehensive profiling of sterols, including the identification of key compounds like β-sitosterol, campesterol [52,65,67], and stigmasterol [83], which are listed in Table 6. β-Sitosterol, constituting 63% of the lipophilic fraction in *C. angustifolium*, is well-known for its cholesterol-lowering properties, making this plant a promising candidate for cardiovascular health supplements [84]. Additionally, its sterol-rich composition supports applications in functional foods and nutraceuticals aimed at regulating lipid metabolism and hormonal balance. Studies by Gryszczyńska et al. further highlight that in vitro cultivation significantly enhances sterol concentrations, with stigmasterol being the most abundant sterol in both in vitro and field-grown samples [52,65,67] (Figure 5).

#### 2.2.6. Tentatively Isolated and Identified Secondary Metabolites from *C. angustifolium*

Frolova and her team conducted an in-depth analysis of the lipophilic components of *C. angustifolium*, with a particular focus on pomolic acid, a bioactive compound exhibiting significant therapeutic potential. Pomolic acid was extracted using methyl tert-butyl ether (MTBE) and further purified through chromatography and recrystallization with a hexane–diethyl ether mixture, yielding 0.04% with a purity of 95%, as confirmed usingchromatography–mass spectrometry (C-MS). In addition, pomolic acid showed notable cytotoxic activity against cancer cells and demonstrated potential in other medicinal applications, including antiviral and anti-inflammatory activities [48].

**Figure 6:**
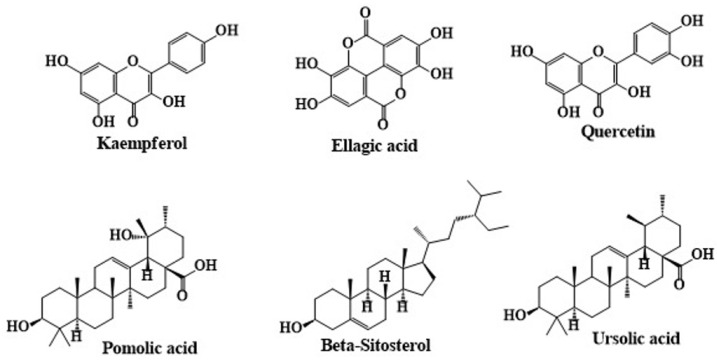
The chemical structures of secondary metabolites isolated from *C. angustifolium*. The structures were drawn using ChemDraw Ultra 12.0 software.

Moreover, Movsumov and his team performed an in-depth study of the biologically active compounds found in *C. angustifolium* cultivated in Azerbaijan. Aerial parts of *C. angustifolium* were collected and air-dried before being extracted with 80% ethanol. The extract was subsequently partitioned with chloroform and ethyl acetate, followed by chromatography using alumina columns, and the structures of the isolated compounds were confirmed using advanced techniques such as nuclear magnetic resonance (NMR) and infrared (IR) spectroscopy. Their research led to the identification of several notable compounds including β-sitosterol, ursolic acid, ellagic acid, kaempferol, and quercetin. Among the identified compounds, β-sitosterol is a plant sterol, while ursolic acid is a pentacyclic triterpenoid. Ellagic acid and the flavonoids kaempferol and quercetin were also detected, each with significant biological activities [85]. The chemical structures of secondary metabolites isolated from *C. angustifolium* are given in Figure 6.

### 2.3. Biological Activity

#### 2.3.1. Diverse Biological Activities of Isolated Compounds from *C. angustifolium*

*C. angustifolium*, a widely recognized medicinal plant, serves as a valuable reservoir of biologically active compounds including pomolic acid, β-sitosterol, ursolic acid, ellagic acid, kaempferol, and quercetin. Among these, quercetin stands out due to its well-documented antioxidant properties, which stem from its ability to neutralize free radicals and prevent lipid peroxidation [42,80,81]. Ulusoy and Sanlier explored the metabolism and bioavailability of quercetin, emphasizing its role in health protection through oxidative stress reduction [86]. Beyond its antioxidant capacity, quercetin has demonstrated significant anti-inflammatory potential by modulating key inflammatory pathways. By inhibiting the release of pro-inflammatory cytokines, such as TNF-α and IL-6, it emerges as a promising therapeutic agent for chronic inflammatory disorders, including rheumatoid arthritis and inflammatory bowel disease [87]. Alizadeh etal. highlighted that the poor water solubility of quercetin poses challenges for its bioavailability, but various delivery systems have been explored to enhance its therapeutic efficacy [88].

Kaempferol, another flavonol present in *C. angustifolium*, has been shown to possess strong antibacterial and antifungal activities. Research conducted by Periferakis et al. demonstrated that kaempferol effectively suppresses the growth of pathogenic microorganisms, including *Staphylococcus aureus*, *Escherichia coli*, and *Candida* spp., reinforcing its potential as a natural antimicrobial agent [89]. In addition to its antimicrobial effects, kaempferol exhibits anti-inflammatory properties through the inhibition of NF-κB activation and the downregulation of COX-2 expression, as described by Alam et al. [90].

Ellagic acid, a polyphenolic compound, has gained considerable attention for its anticancer properties. As demonstrated by Čižmáriková et al., ellagic acid disrupts cancer cell signaling by targeting multiple molecular pathways, thereby inhibiting cell proliferation, angiogenesis, and mechanisms that allow cancer cells to evade apoptosis. Additionally, its chemopreventive role is linked to its ability to enhance the activity of detoxification enzymes and facilitate DNA repair [91].

Ursolic acid, a pentacyclic triterpenoid found in *C. angustifolium*, also exhibits a broad spectrum of biological activities. It functions as a potent inhibitor of inflammatory cytokines and enzymes, such as COX-2 and iNOS, making it a viable candidate for managing inflammatory disorders [92]. Mlala et al. further elaborated on its antimicrobial activity, noting its effectiveness against *Bacillus cereus*, *Escherichia coli*, and *Klebsiella pneumoniae*, supporting its potential use in antimicrobial therapies [93]. Another notable bioactive compound is β-sitosterol, a plant sterol that contributes to cardiovascular health by lowering cholesterol levels through the inhibition of intestinal cholesterol absorption. In addition to its cardioprotective role, β-sitosterol has been linked to immune system modulation, as it enhances T-cell proliferation and reduces inflammation [94].

Finally, pomolic acid, a pentacyclic triterpenoid isolated from *C. angustifolium*, has been extensively studied for its therapeutic potential, particularly in anticancer, antiviral, and anti-inflammatory applications. Research by Martins et al. demonstrated that pomolic acid effectively induces apoptosis and inhibits multidrug resistance mechanisms in prostate cancer cells [95]. Additionally, it exhibits significant cytotoxic effects against lymphocytic leukemia cells and HIV, with an efficacy comparable to 5-fluorouracil. Its mechanism of action includes the stimulation of AMP-dependent protein kinase, the suppression of cancer cell proliferation, and a reduction ininflammation. Importantly, the Ames test and SOS chromotest confirmed its lack of genotoxicity and mutagenicity, supporting its clinical safety profile [48]. Recent studies further highlight pomolic acid’s selective cytotoxicity against glioma cells, including U-87 MG and primary glioma cell lines, demonstrating dose-dependent tumor inhibition [96]. Beyond its anticancer effects, pomolic acid has also shown promise in renal fibrosis treatment. In an in vivo renal fibrosis model, it significantly reduced fibrosis through cadherin and α-SMA modulation while suppressing collagen deposition and extracellular matrix accumulation [97].

#### 2.3.2. Bioactivity of *C. angustifolium* and *C. latifolium* Extracts

##### Antioxidant Properties

The increasing use of plant species for phytotherapeutic applications has driven a surge in studies on their antioxidant properties, as these compounds play a vital role in counteracting oxidative stress [98]. Medicinal plants, abundant in bioactive molecules like phenolic compounds, flavonoids, and terpenoids, are recognized for their strong antioxidant potential [99,100,101,102]. Natural antioxidants have gained prominence as safer and healthier alternatives to synthetic ones [103,104,105].

*C. angustifolium*, a traditional medicinal plant, is distinguished by its abundant phenolic acids, flavonoids, and ellagitannins, which enhance its noteworthy antioxidant qualities [80]. Research indicates that growing methods and solid-phase fermentation (SSF) significantly affect the bioactive chemical composition in fireweed leaves. Evidence indicates that specific SSF parameters enhance the accumulation of certain bioactive compounds in fireweed [80,106]. For example, Lasinskas et al. found that natural fireweed samples exhibited high antioxidant activity (1319.16 M Trolox eq./g D.M.) [76]. Jariene et al. reported that the antioxidant activity initially declined after 24 h of SSF under aerobic (19.23%) and anaerobic (11.14%) conditions but increased after 48 h to levels higher than those of unfermented leaves (324.56 mM TEAC/100 g DW) under aerobic (15.50%) and anaerobic (14.27%) conditions [75].

Additional research by Lasinskas et al. demonstrated annual fluctuations in the antioxidant activity of *C. angustifolium* leaves obtained from a biodynamic farm in Lithuania. In 2017, fermented leaves exhibited more antioxidant activity than unfermented leaves; conversely, in 2018, the reverse was noted. A significant association was observed between the antioxidant activity, quantified in mg 100 g^−1^ DW Trolox equivalents, and the total polyphenol content, with variations dependent on fermentation length [76].

Ecotypes of *C. angustifolium* cultivated in Lithuania showed notable differences in radical scavenging capacity, according to Vilma Kaškonienė et al. [23]. Key bioactive components, such as rutin, caffeic acid, 3,4-dihydroxybenzoic acid, oenothein B, and chlorogenic acid, were found in their study, which also demonstrated a radical scavenging activity ranging from 110.9 to 174.2 mg/g in analyzed samples. These polyphenolic compounds are vital for scavenging free radicals, reducing oxidative stress, and protecting cells from damage due to their potent antioxidant properties. The potent antioxidant capacity of these extracts increases the likelihood of many health benefits, including anti-inflammatory and disease-preventive qualities against oxidative stress [3,107]. The greatest radical scavenging activity was demonstrated by oenothein B in an HPLC-DPPH assay [23].

Maruška et al. evaluated the seasonal radical scavenging activity of *C. angustifolium* in relation to its flavonoid content [66,82]. Leaves harvested at the peak of the blooming phase exhibited the highest flavonoid concentrations (8.71–11.12 mg per 100 g D.M.) and radical scavenging activity [66]. The antioxidant capacity of *C. angustifolium* leaves has been thoroughly investigated, with applications in food preservation and pharmaceutical development examined [108,109].

The antioxidant potential of *C. latifolium* extracts was evaluated usingDPPH radical scavenging and FRAP tests, with both demonstrating significant antioxidant activity. The ethanol extract showed superior activity, with low IC_50_ values of 21.31 ± 0.65 μg/mL (DPPH) and 18.13 ± 0.15 μg/mL (FRAP), underscoring its potential as a natural antioxidant source [9].

##### Antimicrobial Activities

Antimicrobial resistance poses a serious global health threat, driving the search for novel therapeutic alternatives [110]. Increasingly, researchers are turning to plant-derived natural compounds due to their diverse bioactive properties and unique mechanisms of action [111,112,113,114]. Medicinal plants produce a wide range of secondary metabolites, including alkaloids, terpenoids, and phenolic compounds, which have demonstrated potentc antimicrobial activity [115]. The antibacterial properties of *C. latifolium* extracts have also been assessed using the disc diffusion method against Gram-positive (*Staphylococcus aureus*, *Bacillus cereus*), Gram-negative (*Escherichia coli*, *Klebsiella pneumoniae*), and fungal (*Candida albicans*) pathogens. Ethanol extracts exhibited broad activity, with inhibition zone diameters (IZD) ranging from 8.53 to 14.27 mm, whereas ethyl acetate extracts were ineffective against bacteria but inhibited *Candida albicans* (IZD: 8.58 mm) [9]. The antimicrobial effects of plant-derived compounds are often linked to their ability to disrupt cellular membranes, inhibit enzymatic activity, and interfere with essential cellular processes [116]. Additionally, the synergistic interactions between phytochemicals in plant extracts enhance antimicrobial potency and may reduce the risk of resistance development. Several studies highlight the potential of crude plant extracts to work synergistically with conventional antibiotics, improving their efficacy against multidrug-resistant bacteria [117].

##### Anticancer and Cytotoxic Activities

Plant extracts have been widely investigated for their ability to inhibit cancer cell proliferation [118,119]. Maruška et al. reported a dose-dependent suppression of *C. angustifolium* aqueous extracts on breast cancer cell lines (MCF7, MDA-MB-468, and MDA-MB-231), with the most effective concentration ranging from 0.266 to 0.443 mg/mL [66]. The fraction with the highest content of oenothein B (91% phenolics) exhibited the strongest cytotoxic effects, whereas the water-based and third fractions showed comparatively lower activity. Intermediate activity was observed in oenothein B fractions 2 and 3, with the MDA-MB-468 cell line demonstrating the highest sensitivity, indicating the potential of oenothein B in breast cancer therapy [66]. Oenotheins B have been identified as major bioactive constituents in various medicinal plants, particularly within the *Onagraceae*, *Lythraceae*, and *Myrtaceae* families [120]. These macrocyclic ellagitannins are often accompanied by structurally related oligomers, further contributing to their diverse pharmacological properties. Among the most notable biological effects of oenothein B are its antitumor, antioxidant, anti-inflammatory, immunomodulatory, and antimicrobial activities, contributing to its significant health benefits [121]. Traditionally, tannin-rich medicinal plants containing oenothein B have been widely used as folk remedies for various ailments, including gastrointestinal disorders, wound healing, skin conditions, and haemostatic purposes [120], while in vitro and in vivo studies indicate promising anticancer potential. However, further clinical trials are necessary to validate these effects in humans, as factors such as bioavailability, metabolism, and potential side effects must be thoroughly assessed before therapeutic applications can be established.

## 3. Methods

### 3.1. Search Strategy

A comprehensive literature review was conducted to gather relevant scientific data on *C. angustifolium* and *C. latifolium*. The search encompassed main articles published between 2010 and 2024, sourced from major scientific databases including PubMed, Google Scholar, and ScienceDirect. To ensure a thorough and systematic approach, a combination of keywords and Medical Subject Headings (MeSHs) terms was utilized. The search strategy incorporated specific terms related to the plants of interest, such as “*Chamaenerion angustifolium*” and “*Chamaenerionlatifolium*”. Furthermore, additional terms were included to cover various aspects of their phytochemical composition, biological properties, and pharmacological potential. These included “phytochemicals”, “bioactive compounds”, “phytochemical content”, “taxonomic classification”, “botanical description”, “biological properties, activities, or effects”, “pharmacological properties, activities, or effects”, “antioxidant”, “anticancer” or “antiproliferative”, “antidiabetic”, and “antibacterial and antifungal”.

### 3.2. Inclusion and Exclusion Criteria

The inclusion criteria were (I) studies explicitly investigating *C. angustifolium* and *C. latifolium*, including their phytochemical profiles, biological activities, and pharmacological applications; (II) research detailing the identification and quantification of polyphenols, flavonoids, tannins, sterols, and volatile compounds using advanced analytical techniques such as HPLC and GC-MS; (III) studies evaluating antioxidant, antimicrobial, anti-inflammatory, anticancer, and other pharmacological properties; (IV) investigations examining the effects of various extraction techniques (maceration, ultrasonic-assisted extraction, solid-phase extraction) and processing methods (fermentation) on the bioactive compound profile.

The exclusion criteria were (I) short communications, letters, editorials, conference abstracts, and other publications lacking detailed experimental and methodological data; (II) research without a clear focus on *Chamaenerion* species; (III) articles in languages other than English or Russian without a translation.

### 3.3. Data Extraction and Analysis

Relevant studies were selected based on their methodology, experimental design, and reported findings. Extracted data included (I) study type (in vitro research); (II) plant parts used and quantity of material analyzed; (III) analytical techniques applied; (IV) phytochemical composition and identified bioactive compounds; (VI) biological activities and corresponding assays.

Discrepancies in data interpretation were resolved through discussion among the authors.

### 3.4. Addressing Publication Bias

To minimize potential publication bias, the following strategies were implemented:

- Comprehensive database search: inclusion of multiple scientific databases.
- Evaluation of publication trends: analysis of temporal publication patterns to identify biases.
- Assessing methodological consistency: evaluation of study designs and experimental procedures to ensure data reliability.

### 3.5. Study Selection and Quality Assessment

Two independent reviewers (A.K. and Y.T.) assessed the studies for relevance and quality. Disagreements were resolved through discussion. Quality assessment was based on clarity of research objectives, appropriateness of study design, reliability of analytical methods, and consistency of reported results.

### 3.6. Screening and Selection of Relevant Studies

An extensive database search initially identified 2010 studies. After screening based on the established inclusion and exclusion criteria, 52 articles were selected for data extraction and results analysis. The discussion of these findings is provided in the subsequent section.

## 4. Conclusions and Future Perspectives

This review underscores the exceptional phytochemical diversity and pharmacological potential of *C. angustifolium* and *C. latifolium*. These species are notable for their abundance of bioactive compounds, including polyphenols, flavonoids, ellagitannins, and volatile constituents, which are closely associated with their pronounced antioxidant, anti-inflammatory, antimicrobial, and anticancer activities. Utilizing advanced analytical methodologies, such as GC-MS and HPLC, researchers have gained valuable insights into their chemical compositions, laying a solid foundation for their diverse therapeutical applications.

A strong correlation exists between the biological activities of *Chamaenerion* species and their chemical composition. Polyphenols such as oenothein B and flavonoids like quercetin contribute significantly to their antioxidant and anti-inflammatory effects. Ellagitannins exhibit potent anticancer potential, while volatile compounds like α-pinene and linalool enhance antimicrobial effects. The influence of factors such as fermentation, environmental conditions, and extraction techniques has been extensively investigated, revealing significant modifications in the composition and bioavailability of these bioactive compounds. Among these factors, fermentation stands out as it enhances the concentration of polyphenols and flavonoids, thereby amplifying the antioxidant and anti-inflammatory properties of *Chamaenerion* species.

Nevertheless, despite considerable advancements, notable gaps in the existing knowledge persist. Future research should prioritize the following:

Optimizing extraction and processing techniques to maximize the yield, stability, and efficacy of bioactive compounds.

Elucidating the synergistic interactions of *Chamaenerion* compounds within complex biological pathways.

Conducting rigorous clinical trials to confirm their therapeutic efficacy and safety for human health.

The integration of traditional medicinal knowledge with contemporary scientific approaches presents an exciting avenue for fully realizing the medicinal potential of *Chamaenerion* species. Continued interdisciplinary investigations will not only enhance our understanding of their chemical and pharmacological attributes but also facilitate the development of innovative health-promoting products, addressing the rising global demand for natural and sustainable therapeutic solutions.

## Figures and Tables

**Table 1:** Taxonomic classification of *C. angustifolium* and *C. latifolium*.

Kingdom	Plantae
Family	*Onagraceae*
Phylum	Tracheophyta
Class	Magnoliopsida
Order	Myrtales
Subfamilies	Onagroideae
Genus	Oenothera
Species	*Chamaenerion angustifolium* *Chamaenerion latifolium*

**Table 6:** Sterols identified in *C. angustifolium* extracts.

Compounds	Molecular Weight, g/mol	Identification Method	Extraction Method	Extract Type	Ref.
Sterols					
Campesterol	400.69	HPLC-DAD, GC-MS	Percolation, UAE	Methanol; MTBE	[52,65,67]
Stigmasterol	412.70	HPLC-DAD, GC-MS	Percolation, SPE, UAE	Methanol; MTBE	[52,65,67]
β-sitosterol	414.72	HPLC-DAD, GC-MS	Percolation, UAE	Methanol; MTBE	[52,65,67]

MTBE—methyl tert-butyl ether; UAE—ultrasonic-assisted extraction; SPE—solid-phase extraction.

## Data Availability

No new data were created or analyzed in this study.

## Abbreviations

The following abbreviations are used in this manuscript:

5-LOX	5-Lipoxygenase
α-SMA	Alpha-Smooth Muscle Actin
AMP	Adenosine Monophosphate
BMI	Body Mass Index
*CA*; *C. angustifolium*	*Chamaenerion angustifolium*
*CL*; *C. latifolium*	*Chamaenerion latifolium*
COX-2	Cyclooxygenase-2
C-MS	Chromatography-Mass Spectrometry
DNA	Deoxyribonucleic Acid
DPPH	2,2-Diphenyl-1-picrylhydrazyl
DM	Dry Mass
DW	Dry Weight
FRAP	Ferric Reducing Antioxidant Power
GC-MS	Gas Chromatography-Mass Spectrometry
HPLC	High-Performance Liquid Chromatography
HPLC-DAD	High-Performance Liquid Chromatography with Diode Array Detection
HPLC-DAD-MSn	High-Performance Liquid Chromatography with Diode Array Detection and Multi-Stage Mass Spectrometry
HPLC-UV	High-Performance Liquid Chromatography with Ultraviolet Detection
HPLC-UV-ESI/MS	High-Performance Liquid Chromatography with Ultraviolet Detection and Electrospray Ionization Mass Spectrometry
IC_50_	Half-Maximal Inhibitory Concentration
IL-6	Interleukin-6
iNOS	Inducible Nitric Oxide Synthase
IR	Infrared Spectroscopy
IZD	Inhibition Zone Diameters
MCF7	Michigan Cancer Foundation-7 (Human Breast Cancer Cell Line)
MDA-MB-231	Human Triple-Negative Breast Cancer Cell Line
MDA-MB-468	Human Triple-Negative Breast Cancer Cell Line
MeSH	Medical Subject Headings
mPGES1	Microsomal Prostaglandin E Synthase-1
MTBE	Methyl tert-butyl ether
NMR	Nuclear Magnetic Resonance
NF-κB	Nuclear Factor Kappa B
PC	Paper Chromatography
PMs	Primary Metabolites
Ref.	References
SPME	Solid-Phase Microextraction
SSF	Solid-Phase Fermentation
TEAC	Trolox Equivalent Antioxidant Capacity
T-cell	T Lymphocyte (a type of immune cell)
TNF-α	Tumor Necrosis Factor Alpha
U-87 MG	Human Glioblastoma Cell Line
UAE	Ultrasonic-Assisted Extraction
